# Experience of Playing Sport or Exercising for Women with Pelvic Floor Symptoms: A Qualitative Study

**DOI:** 10.1186/s40798-023-00565-9

**Published:** 2023-04-25

**Authors:** Jodie G. Dakic, Jean Hay-Smith, Kuan-Yin Lin, Jill Cook, Helena C. Frawley

**Affiliations:** 1 Department of Physiotherapy, Monash University, 47 - 49 Moorooduc Highway Frankston, Victoria, 3199 Australia; 2 Rehabilitation Teaching and Research Unit, Department of Medicine, University of Otago, Wellington, PO Box 7343, Wellington South, 6242 New Zealand; 3 Department of Physical Therapy, National Cheng Kung University, No. 1, Ta-Hsueh Road, Tainan, 701 Taiwan; 4 Institute of Allied Health Sciences, College of Medicine, National Cheng Kung University, No. 1, Ta-Hsueh Road, Tainan, 701 Taiwan; 5 La Trobe Sport and Exercise Medicine Research Centre, La Trobe University, Plenty Rd &, Kingsbury Dr, Bundoora, VIC 3086 Australia; 6 Melbourne School of Health Sciences, The University of Melbourne, 161 Barry St., Parkville 3010, VIC Australia; 7 Allied Health Research, The Royal Women’s Hospital, Parkville, VIC 3052 Australia; 8 Allied Health Research, Mercy Hospital for Women, Studley Rd, Heidelberg, VIC 3084 Australia; 9 Department of Physiotherapy, The University of Melbourne, 161 Barry St, Parkville, 3010 Australia

**Keywords:** Pelvic pain, Anal incontinence, Urinary leakage, Pelvic organ prolapse, Physical activity, Female, Sportswomen, Experiences, Pelvic floor dysfunction

## Abstract

**Background:**

Women participate in sport at lower rates than men, and face unique challenges to participation. One in three women across all sports experience pelvic floor (PF) symptoms such as urinary incontinence during training/competition. There is a dearth of qualitative literature on women’s experiences of playing sport/exercising with PF symptoms. The purpose of this study was to explore the lived experience of symptomatic women within sports/exercise settings and the impact of PF symptoms on sports/exercise participation using in-depth semi-structured interviews.

**Results:**

Twenty-three women (age 26–61 years) who had experienced a breadth of PF symptom type, severity and bother during sport/exercise participated in one–one interviews. Women played a variety of sports and levels of participation. Qualitative content analysis was applied leading to identification of four main themes: (1) I can’t exercise the way I would like to (2) it affects my emotional and social well-being, (3) where I exercise affects my experience and (4) there is so much planning to be able to exercise. Women reported extensive impact on their ability to participate in their preferred type, intensity and frequency of exercise. Women experienced judgement from others, anger, fear of symptoms becoming known and isolation from teams/group exercise settings as a consequence of symptoms. Meticulous and restrictive coping strategies were needed to limit symptom provocation during exercise, including limiting fluid intake and careful consideration of clothing/containment options.

**Conclusion:**

Experiencing PF symptoms during sport/exercise caused considerable limitation to participation. Generation of negative emotions and pain-staking coping strategies to avoid symptoms, limited the social and mental health benefits typically associated with sport/exercise in symptomatic women. The culture of the sporting environment influenced whether women continued or ceased exercising. In order to promote women’s participation in sport, co-designed strategies for (1) screening and management of PF symptoms and (2) promotion of a supportive and inclusive culture within sports/exercise settings are needed.

**Supplementary Information:**

The online version contains supplementary material available at 10.1186/s40798-023-00565-9.

## Key Points

- Women with PF symptoms experienced a substantial impact on their ability to participate in sports/exercise, leading to cessation or limitations to intensity, frequency or mode of involvement. Provision of evidence-based management of PF symptoms may allow women the opportunity to continue participation in sports/exercise with less impact.
- Experiencing PF symptoms during sport/exercise led to feelings of embarrassment, frustration and fear of symptoms becoming known to others with subsequent avoidance of group settings. Co-designed and acceptable screening for PF symptoms within sports/exercise settings, may allow women the opportunity to disclose symptoms, seek help and enhance participation in organised sport/exercise.
- The culture of sports/exercise settings influenced whether women felt supported to continue exercising or chose to stop participation; positive role models (other members or support staff), education and re-design of uniform to allow covert containment of leakage were strategies identified to promote a positive culture.

**Supplementary Information:**

The online version contains supplementary material available at 10.1186/s40798-023-00565-9.

## Background

Physical inactivity is a major risk factor for early mortality, chronic illness and poor mental health [1]. In 2019, only 14% of adult Australian women met the National Guidelines for physical activity and muscle strengthening exercise [2]. Women face several unique barriers to maintaining physical activity across their lifespan including childcare/carer responsibilities, lack of time or energy and concerns about body image and personal security [3, 4]. However, for women with a pelvic floor (PF) disorder, their symptoms are often an additional exercise barrier. A previous study by our group, of 4556 Australian, adult, women with PF symptoms, found that one in two stopped a form of sport/exercise secondary to their symptoms [5]. One in three younger (18–25 years) and nulliparous women reported PF symptoms caused them to cease a form of sport/exercise [5]. One in three symptomatic women found their symptoms to be a substantial barrier causing them to stop sport/exercise often/all of the time [6]. Women with severe urinary incontinence (UI) were at greater odds of reporting sport/exercise cessation than those experiencing moderate/mild symptoms (OR = 4.77;95% CI = 3.60–6.34) [6]. However, even for women who reported mild severity, one in four women had stopped a form of exercise [5]. Women who identified their PF symptoms as a substantial barrier to exercise were also at greater odds of physical inactivity [6]. Thus, experiencing PF symptoms has a negative impact on exercise participation.

During sport/exercise the PF must counteract the increase in intrabdominal pressure and ground reaction forces generated from high-impact activities and heavy lifting. Symptoms may be provoked during sport/exercise if PF function is suboptimal [7]. Pelvic floor symptoms are highly prevalent in female athletes and exercising women [8, 9]. On average, 1 in 3 women participating in sport have experienced symptoms of UI.[9], including 50% of adolescent athletes (< 19 years) [10]. The prevalence rises to 80% in trampolinists/gymnasts [9, 11, 12] and approximately 50% in women participating in high-impact, ball sports such as basketball [8], rugby [13] and tennis [8]. Beyond UI, less data exist on the prevalence of other types of PF disorders. Athletes experience anal incontinence [AI] (leaking wind or stool), with prevalence rates of 80% in power/weight lifters [14], 80% in gymnasts [12] and 28% in triathletes (28%) [15]. One in four power/weight lifters report symptoms of pelvic organ prolapse (POP) [14].

Self-disclosure of PF symptoms by female athletes is uncommon; less than 10% have ever told a health professional [16–19]. A recent systematic review of quantitative data found that elite female athletes employed coping strategies such as wearing pads, restricting fluids and frequent voiding to manage symptoms of UI [20].

There is a lack of in-depth knowledge on the experience of playing sport/exercising with PF symptoms. A recent systematic review by our group found limited qualitative data on the effect of PF symptoms on sport/exercise participation and most studies were investigating the influence of PF symptoms on overall quality of life (QoL) rather than effect on exercise directly, therefore, data were scarce and not detailed [21]. In a study investigating the impact of UI on QOL for women living in China (*n* = 15), women avoided participating in social activities, i.e. walking or climbing mountains, which caused feelings of emotional isolation. Other studies investigating the effect of UI symptoms on QOL have recruited primarily from populations with specific impairments that would impact broadly on exercise participation (gynaecological cancer survivors [22], newly post-natal mothers [23] and women > 65 years with urgency UI [24]) and may therefore not represent the experience of exercising with UI in the broader population. Our systematic review found only one study with the primary purpose of understanding 18–65 years old women’s experience of UI symptom impact on exercise. [25]**.** This mixed-methods study, in high-impact recreational athletes (18–45 years), included a single focus group (*n* = 7) and found that athletes felt worried, frustrated and fearful of provoking symptoms [18].

Beyond UI, the qualitative literature on how experiencing other types of PF symptoms impact women’s ability to play sport/exercise is even more sparse. In two qualitative studies of women’s general life experience of POP symptoms, participants reported feelings of frustration, social isolation and concerns about exercise worsening symptoms, particularly in the post-natal period [26, 27]. There have been no qualitative studies exploring the impact of AI on sports/exercise participation [21].

Quantitative data have established that many women stop or adapt their exercise participation secondary to PF symptoms. Focused qualitative research will allow an understanding of the psychosocial and environmental factors that influence the decisions women make regarding modifications and planning for exercise, secondary to their symptoms. An understanding of the challenges symptomatic women experience in sports/exercise settings will aid development of strategies to create a supportive and safe environment for women to exercise with PF symptoms.

The purpose of this study was to explore women’s experiences of playing sport/exercising with PF symptoms and the impact PF symptoms have on sports/exercise participation.

## Methods

### Study Design

A qualitative descriptive design was used. Qualitative description is commonly used in health science research to explore the experience within a setting/organisation (in this case women who have experienced PF symptoms whilst exercising in sports/exercise organisations) [28]. Qualitative descriptive methods aim to provide a detailed account (stay close to the data) [29], of participants’ perspectives and experiences in everyday language, that can be readily understood by the broader community they represent [28, 29]. This approach has been used by two prior studies exploring the experience of pregnant/post-natal female athletes within sports/exercise settings [30, 31]. This study was approved by the University of Melbourne Human Research Ethics Committee (Ref - 2021-22466-21024-3, 24th August 2021). Participants provided written consent and reconfirmed verbally at the start of the interview. This study is reported according to the Consolidated Criteria Of Reporting Qualitative Studies (COREQ) guidelines [32] and the Standards for Reporting Qualitative Research (SRQR) guidelines [33].

### Participants

Participants were 18–65-year-old women, residing in Australia who had experienced PF symptoms whilst exercising (in the past or currently). Participants were recruited from an existing database of women identified during a prior quantitative survey study [5, 6], who had agreed to be contacted via email with an invitation to participate in further research. In groups of 30, participants from the database were sent an email invitation to participate in the study. After 11 interviews were completed, we assessed whether our sample had participants with a broad range of qualities or experiences representative of exercising women who experience PF symptoms [29, 34]. Characteristics we agreed (and defined a priori) might generate quite different experiences of the impact of PF muscle symptoms on their exercise/sport were: age; parity; level of participation in sport when PF symptoms were first experienced; PF symptom type/severity/bother and disclosure of PF symptoms to a health/exercise professional in a sports/exercise setting (yes/no). Younger, nulliparous, and elite/semi-elite athletes were under-represented, so an additional sentence in the email invitation was added stating we would particularly like to hear from women in these groups.

The email contained an invitation to the study, a plain language statement and a link to a short (< 5 min) Qualtrics survey. The Qualtrics survey contained questions to determine: consent (yes/no), eligibility (age, current postcode in Australia, experience of PF symptoms whilst exercising) and questions that helped us assess representativeness of the sample. Age was classified according to lifespan periods where PF symptom onset commonly occurs, pre-childbearing years (18–25 years), childbearing (26–45 years), menopausal (46–55 years) and post-menopausal (56–65 years) [35]. Validated (Grade A, ICI recommended) [36] PF questionnaires were also included to determine: urinary incontinence (UI) prevalence and type (Questionnaire for Urinary Incontinence Diagnosis [37]); UI severity (Incontinence Severity Index [38]); UI bother (Pelvic Floor Bother Questionnaire [39]) and anal incontinence (AI) and pelvic organ prolapse (POP) prevalence and bother (Pelvic Floor Bother Questionnaire [39]). Participants received a $30 gift card as acknowledgement of their participation and time spent in the interview.

Sample size was guided by the concept of information power, which is recommended for qualitative descriptive research [40–42]. Factors that evaluated when our study had generated sufficient depth and breadth of data (information power) were: (1) a relatively narrow aim with a targeted and representative sample of participants with experience of the phenomenon being investigated (women’s perceptions of the experience and disclosure of PF symptoms specifically within sports/exercise settings) [40, 41] (2) the purpose of the study that aimed to describe an experience rather than generate a theory [40] (3) one-to-one interviews of average length 40 min, using open-ended questions that were adapted in an iterative manner allowing for generation of rich data [40, 41] and (4) exploratory analysis—utilising patterns to stay close to the participants’ descriptions of their experience [40, 41].

### Interview Design

This study was part of a larger parent mixed-methods project that explored exercising women’s experiences of PF symptom disclosure, screening and management within sports/exercise settings. A semi-structured interview schedule with open-ended questions was developed using knowledge generated from a previously published systematic review by our group [21]. Questions were kept explorative and open-ended in order to encourage participant-led discussion. The two key questions from the interview schedule specific to the aims of this study were ‘What sort of exercise and physical activity are you doing at the moment/have done in the past?’ and ‘What effect are PF symptoms having/previously had on your exercise?’ Prompts (such as types of exercise, impact of different PF symptoms type, nature of impact and adaptations made if exercise was continued) were used to elicit detailed descriptions. The interview schedule was piloted with a female key informant with experience of PF symptoms during exercise, prior to the study to allow refinement of the question schedule. A summary of research topics to be discussed was sent to participants prior to the interview (Additional file 1).

### Data Collection

Interviews occurred between November 2021 and March 2022 via Zoom online platform with video enabled. All interviews were conducted by one researcher JD (female, experienced physiotherapist practising in sports and PF physiotherapy, academic and PhD candidate who had undergone training in conducting qualitative interviews). The interviewer (JD) did not know any of the participants prior to the interview and participants were not informed of the researcher’s occupation/background prior to or during the interview. Participants were informed that this project was a follow-up study from part of a larger project that they had previously participated in via an online anonymous survey. Interviews were recorded (audio only), transcribed verbatim by a professional medical transcriptionist and checked for accuracy against the audio by a researcher (JD). The length of the interviews ranged from 20 to 63 min (average length 40 min).

A one-page descriptive summary of the woman’s interview (main thoughts and feelings) was written and sent to her for comment/correction. The interviewer (JD) kept a research journal, completed after each interview and throughout analysis. Reflections included interview behaviours, schedule, length of interview, confidence in attaining reliable information, interactions with participants, and the researcher’s role and influence on the data. Reflections were used to adapt the interview guide in an iterative manner as data continued to be collected, and throughout data analysis to explore researcher assumptions and stay as near to the participants’ meanings as possible [43].

### Data Analysis

Data were analysed using qualitative content analysis [44]. Data were managed using NVivo software. Deidentified transcripts were read in entirety initially, for familiarisation with the data as a whole [43, 45]. Inductive content analysis was used to identify and note key concepts or similarities within the data [28]. Commonalities or patterns within the data led to development of a coding scheme with definitions, that was then applied to all data [45]. Codes were merged or linked to form themes representing the latent meaning of the content [44, 45]. One researcher (JD) coded all data and developed the initial coding tree. Members of the research team (JHS, HF and KYL) also independently coded a selection of transcripts. Additional codes, and coding differences, between researchers were discussed by the research team until consensus. The team met fortnightly until final themes were agreed. Participant quotations were selected to illustrate themes [44]. Quotations were altered by removing a few words without altering the meaning; where this has occurred, it is indicated by three consecutive ellipses. Following data analysis, a written summary of the main findings of the project was sent to participants and they were invited to comment on the findings [44].

## Results

A total of 309 participants from an existing database were contacted via email. Forty-one women, engaged with the survey link in the email (read the plain language summary), one declined participation; three participants did not complete the survey to determine eligibility and one was excluded after completing the survey (age > 65 years). Thirty-six participants consented, completed the survey and met the inclusion criteria. However, eleven participants did not respond to two approaches to arrange an interview time, and data from one participant (P5) were excluded from analysis because (contrary to the screening questionnaire data) at the interview she clearly stated she had not experienced past or current PF symptoms during exercise.

The 23 women were an average age of 46.2 years (range: 26–61 years), 87% were parous and 78% played sport at a recreational level. Pelvic floor symptom prevalence was 96% UI, 35% POP and 48% AI (see Table 1 for summary of participant characteristics). The findings of this study are presented in four themes: (1) I can’t exercise the way I would like to (2) it affects my emotional and social well-being, (3) where I exercise affects my experience and (4) there is so much planning to be able to exercise.

**Table 1:** Characteristics of participants

P	Age category (years)	Level of sports participation when PF symptoms first experienced	Disclosure of PF symptoms to H/E professional in sports/exercise settings	Parous	No. of vaginal births	Presence of:						Severity	Bother		
						UI^†^	SUI^‡^	UUI^‡^	MUI^‡^	AI	POP	UI	UI	AI	POP
1	46–55	Social/non-competitive	Y	Y	3	Y			Y		Y	Moderate	Moderately		A lot
2	26–45	Social/non-competitive	Y	Y	2	Y				Y	Y	Slight	Only a little	Somewhat	Somewhat
3	26–45	Elite	Y	Y	1	Y	Y					Moderate	Somewhat		
4	46–55	Local club/school team	N	Y	3	Y	Y					Moderate	A lot		
6	56–65	Social/non-competitive	N	Y	1	Y	Y			Y	Y	Moderate	Somewhat	Somewhat	Somewhat
7	56–65	Social/non-competitive	N	Y	2	Y			Y			Moderate	Moderately		
8	26–45	Social/non-competitive	N	Y	3	Y			Y	Y	Y	Moderate	A lot	A lot	A lot
9	26–45	Social/non-competitive	Y	Y	1	Y	Y					Moderate	A lot		
10	26–45	Local club/school team	Y	N	0					Y			Not at all	Not at all	
11	56–65	Social/non-competitive	N	Y	2	Y						Slight	Moderately		
12	56–65	Social/non-competitive	Y	Y	2	Y			Y	Y	Y	Slight	Somewhat	Somewhat	Somewhat
13	46–55	Social/non-competitive	Y	Y	0	Y	Y			Y		Moderate	Moderately	Moderately	
14	26–45	Social/non-competitive	N	Y	1	Y			Y	Y	Y	Severe	Somewhat	Moderately	Moderately
15	26–45	Social/non-competitive	Y	Y	2	Y	Y				Y	Severe	A lot		A lot
16	26–45	Social/non-competitive	Y	Y	2	Y			Y			Severe	A lot		
17	46–55	Social/non-competitive	N	Y	1	Y	Y			Y		Moderate	Somewhat	Only a little	
18	46–55	Social/non-competitive	Y	Y	2	Y			Y	Y		V. severe	A lot	A lot	
19	26–45	Social/non-competitive	N	N	0	Y						Moderate	Somewhat		
20	46–55	Local club/school team	N	Y	1	Y	Y					Moderate	Somewhat		
21	26–45	Sub-Elite	N	Y	0	Y			Y			Moderate	Somewhat		
22	56–65	Social/non-competitive	Y	Y	2	Y	Y			Y		Slight	Only a little	Only a little	
23	26–45	Social/non-competitive	N	N	0	Y			Y	Y		Moderate	Somewhat	Somewhat	
24	26–45	Social/non-competitive	Y	Y	1	Y	Y				Y	Moderate	Somewhat		Only a little

*P*—Participant, *PF*—pelvic floor, *H*/*E*—health/exercise, *UI*—urinary incontinence, *SUI*—stress urinary incontinence, *UUI*—urgency urinary incontinence, *MUI*—mixed urinary incontinence, *AI*—anal incontinence, *POP*—pelvic organ prolapse. Elite level of sports participation = competed at international or national level, Sub-Elite = competed at state or district level of competition

^†^Defined as report of any urinary leakage symptom on QUID

^‡^As per summed score on questionnaire for urinary incontinence diagnosis

### I Can’t Exercise the Way I Would Like to

Women spoke of the extensive impact that their PF symptoms had on their ability to exercise. Women ceased playing a variety of sports secondary to their symptoms including high-impact: netball, running, high-intensity interval training exercise class, abseiling and pole-dancing and low-impact: walking and swimming (e.g. *“I don't run. If I run, I’m in trouble.” [P1]).* Women gave many examples of specific exercise activities or movements that provoked PF symptoms:

*“I can't get into a low squat because I'll leak. Skipping on a rope. Anything that requires me to go up and down basically, as soon as I move like that, my pelvic floor fails, and I struggle with sit ups as well.” (Participant [P]16).*

One way to deal with symptoms was to modify their exercise by changing from high to a low-impact form of exercise (e.g. “*in any of the classes where there's a skipping or high knee, I don't do it.” [P24]*). Other strategies were lowering the intensity of exercise (e.g. *“it's quite limiting with respect to the intensity that you can actually put into what you do… quite often just in the warm up because we're doing sprints and star jumps” [P4]*), selecting the time of the day (e.g. avoiding afternoons when symptoms were worse), or avoiding exercise at certain stage of their menstrual cycle.

Women also titrated the amount of time and effort they put into training or competition because that influenced the expression of their symptoms, or symptom severity.

*“If you feel it coming on and you can feel … there's potential for wetness there, that you will pull back a bit and not go as hard as you would've gone otherwise.” (P19)*

If symptoms continued to be bothersome or deteriorated despite exercise modification strategies, women might choose to cease organised sport/formal exercise entirely. A number of women reported feeling restricted to short walks on a flat/even surface as their only exercise option.

*“I used to play netball…Because it happens when you're in the middle of sport…being anal leakage, because that's something I struggle a lot with. Obviously, the more you exercise and the more you do sport and things, it flares up, but it gets really embarrassing where you don't actually want to do anything and hang out with anyone.” (P14).*

*“Can't really handle long walks, because I need to often use the bathroom.” (P23)*

Group exercise environments, such as a gym or exercise class, concerned women. A fear of others noticing symptoms through leakage (flatus, faecal matter, urine) limited exercise choices. *“I really would like to join a gym, but I worry, and I have to wear a pad all the time. Well, sometimes, even though I've got a pad in, and I have the longest ones on the market, sometimes it squeezes over. And then of course, because I also have the issue of bowel leakage, of wind, I can't win.” (P18).*

Impact on sport/exercise participation was experienced amongst women with all degrees of UI severity. Avoidance/modification of high-impact activities, endurance exercise and heavy lifting was commonly reported amongst women with slight symptom severity (via Incontinence Severity Index[38]). Additionally, women with severe/very severe UI symptoms reported also avoiding low-impact forms of exercise e.g. swimming, hydrotherapy and walking quickly or on uneven ground.

Leakage was not the only PF symptoms of concern. Symptoms consistent with POP, e.g. feeling of dragging in the vagina or lower abdomen, caused women to stop participating. *“Doing things like boot camps…I just felt like underneath was just going to fall out.” [P8].* Women with POP symptoms reported avoiding running and lifting heavy weights. They limited exercise later in the day when symptoms tended to be worse. Pelvic pain also necessitated modification of exercise or additional pain management techniques (e.g. *“If I'm having a pain flare … I end up staying at the gym for about half an hour afterwards with a heat pack on just to get it back to normal before I drive home.” [P10]).*

*“With the gym now, if I'm having a bad flare, I get pain shooting through my pelvis and shooting through my rectum and shooting through my cervix. So usually I will either try and push through or I will modify the exercise to try and release some of the pain and discomfort.” (P24).*

### It Affects My Emotional and Social Well-Being

Women were fearful that someone would notice their PF symptoms; they worried people could see the leakage on clothing, and hear/smell leakage associated with AI. Women found POP symptoms easier to hide from other exercisers *“the heaviness, that happens as well, but nobody knows about that.” (P20)* However, women felt that others within sports/exercise settings were less familiar with providing support for those experiencing POP symptoms *“so he (personal trainer) was really good, but you could also tell he didn't get it. It's hard to understand, … because I have a prolapse and so it's quite difficult to understand that” (P2).*

They found it frustrating that exercise was less enjoyable because of all the ‘rules’ they had to follow to try and avoid provoking symptoms. Women expressed frustration and bother at the level of planning, problem solving and attentiveness required to exercise*. “I know that there's the potential for leakage and so it's in the back of my mind whenever I'm exercising.” (P6)* Feeling overwhelmed or de-motivated by this led to exercise cessation when coupled with all the other barriers to exercise women commonly faced.

*“Why can’t I run in the afternoon? Why do I have to run in the morning? Why do I have to be prepared for everything and think it all through before I do it? I don't need barriers to exercise, I already have enough trouble with my own motivation levels.” (P9).*

Participants found it difficult to be spontaneous and exercise at a convenient time or in their preferred way. They often felt like they were missing out on enjoying the fun of exercising with family/friends or joining in impromptu family games. *“It frustrates me on so many different levels. There's just the fact that you can't kind of be spontaneous. If the kids are doing something, and they're like, "Do you want to play tiggy?" And I'm like, "Oh, I'd really like to, but sadly I'm going to say no, because I don't want to embarrass myself and wet my pants.” (P9).*

Women were embarrassed and ashamed of their PF symptoms. They worried others were looking at them or judging them. This was especially apparent for younger women and those who had not experienced childbirth. The belief that symptoms were only experienced by post-natal women heightened feeling of stigma and embarrassment for nulliparous women.

*“I just was embarrassed. I never wanted to tell anyone. I didn't want anyone to know. It was like a dirty little secret. That I was young. I hadn't had kids and I still leaked, so it comes with the judgment and the rest of it” (P16).*

Women expressed concerns of how other exercisers perceive them within sports/exercise settings particularly if they were unable to participate in some activities within an exercise session due to their symptoms (e.g. “*Don't think that I'm being lazy, I'm having a medical thing going on.” [P23])* Others had experienced being isolated from the group setting because modifications to exercise were not included or able to be demonstrated. Women found that when seeking exercise modifications, they had to decide between the social consequences of symptom disclosure versus wanting to keep their condition private. Women found it difficult to build rapport with their health/exercise professionals when they chose not to disclose their symptoms, worrying that the exercise instructor was judging their effort or commitment.

*“Because I know that if I'd started out and they said, "Okay, let's skip rope," you would hear, "No, I'm not doing that." And then they just thought you were lazy. It'd just shift the relationship that the two of you would possibly have.” (P16).*

### Where I Exercise Affects My Experience

Participants considered the different sports/exercise cultures they had been involved in and the impact of these on their experience of exercising with PF symptoms. The extent of trust and rapport in the sporting community were important to how comfortable women felt exercising with PF symptoms.

Sporting environments composed of predominantly male members or masculine culture were less comfortable. Participants felt men would not understand the nature of their symptoms, not be interested in hearing about them and participants felt embarrassed talking about their symptoms or requesting modifications to exercise when males were present.

*“I know a lot of women feel uncomfortable about working out where there are men around as well, so they like the women only spaces and stuff. If you're going into somewhere that's not like that, I can imagine that you would feel very self-conscious about people looking at you and you don't want to give people an extra reason to look at you and you also don't like to think of the PTs going and talking to all of their colleagues about you.” (P10).*

A positive culture for women with PF symptoms was encouraged when other exercisers/athletes openly discussed their PF symptoms within the group, making women feel less isolated and normalising the conversation. For instance, one woman was a power lifter and said the leakage was so common in that setting that “*it's much, much easier to bring it up because women will see it happening to other women” (P10).* In contrast, the strict disciplines and etiquette of the dance environment made it hard for another participant to exercise in her preferred way “*because you were supposed to look a certain way, you were supposed to able to do certain things."(P16)* Professional athletes could contribute to a positive culture and confidence to disclose, discuss and pursue solutions.

*“If you saw a professional sports person or even an amateur sports person who was open about their experience. If it was something that they were willing to share, that would definitely help. There's an easy way then that, you could go say to someone like, "Hey, I was just going and watching this … at the Olympics. And they were saying that they've struggled with this. I was so surprised". And then that person might say, "Oh, hey, I've actually had that too. I didn't realize that someone so young would also experience that" (P19).*

It was harder for women to discuss their symptoms in sports organisation that were larger or lacking personal connections/relationships. One participant spoke of her preference for smaller, personalised exercise environments with fewer members: “*if that were to happen in the big box gym, I'd feel super embarrassed and just go home, but here I'd feel comfortable enough to shower and freshen up. I guess the space to feel as comfortable as possible.” (P23).*

Another participant expressed that although her sports team has a very close relationship, ‘taboo’ topics such as health conditions are not discussed between team members *“because we are more so focused on what we're trying to get done and we don't generally chit chat a lot about medical things. It's more a taboo subject really. We don't really talk about our medical problems to anyone. We're a close team but we still don't talk about medical issues really.” (P21)* Participants felt that marketing and exercise programmes at sports/exercise organisations did not recognise common health conditions such as PF symptoms and did not account for older women, which made it hard to feel comfortable to exercise with symptoms.

*“To be able to get more women of a certain age, then the material they need to market to those women needs to include information about common issues, I suppose that they experience. Then we would feel more comfortable going if that information was available.” (P24).*

### There is so Much Planning to be Able to Exercise

Women managed symptoms through implementing meticulous coping strategies and monitoring symptoms with vigilance, which reduced spontaneity and carefree exercise participation. Coping strategies required planning in advance (e.g. limiting fluids and carrying extra clothing) or constant attention (not exercising too far from toilets or being aware of odour/visible leakage). (Table 2).

**Table 2:** Coping strategies for exercising with pelvic floor symptoms

Coping strategy	Supporting quotation
**Toileting** Emptying the bladder multiple times during or prior to exercise Ensuring that run/walk routes were always near a toilet Sourcing facility with good access to toilets, i.e. changing to a smaller gym where the toilet is closer to the class	*“Well I'm usually pretty careful. If I was going to a live yoga class, if I had to travel a distance to get to the yoga class, I go to the toilet before I get on the mat.” (P11)* *“I think being so accessible to the bathroom, that's the main thing for me. I think being kept at kind of small class groups, it makes it more comfortable to not think that I'm hogging the toilet or anything.” (P23)*
**Containment** Wearing and frequently changing pads/underwear Taking a change of clothes for exercise in case of accidental leakage	*“So, you're quietly going to the toilet, change your underwear again, you take three pair of underwear and a plastic bag and what not. You buy those special little wipes that you can buy to clean up on each time. But I was sort of hyper aware because I was in a hot and sweaty environment” (P3)*
**Concerns about clothing/uniforms** Being conscious of clothing/uniform selection for sport/exercise Stopping sports when uniforms do not allow for concealment of pads or leakage e.g. short, light-weight, cut-away or light-coloured outfits made women fearful that leakage could be seen and made it difficult to wear pads or continence underwear Choosing to wear dark clothing that can hide leakage Feeling of distress when sports (like martial arts) required white uniforms where leakage is more visible	*“It bothers me because I have to wear black tights. I'd like to wear some lovely coloured tights that women can wear, but I just can't.” (P24)* *“For some women where pads don't hold it, then they're going to need that extra support because they're likely to have more of an issue of leakage at a sport setting. Especially netball ones. They're quite short.” (P14)*
**Intake restrictions** Restricting fluid intake before and during sport/exercise to avoid a full bladder Balancing adequate hydration with avoiding leakage. Concerns about dehydration particularly in hot climates Women with anal incontinence also carefully considered the timing of the intake of food and medications to avoid provoking bowel leakage	*“I deliberately don't drink, which is probably a bit of a thing, … before I'm going to go for a run.” (P13)* *“I think for me mostly it's like urine incontinence, especially after a hard run. I know if I'm pushing myself or if the weather is particularly hot. I notice the impacts then, especially through summer, where you're trying to stay hydrated, but at the same time being mindful of… Well, I need to make sure that I balance this out too and not wet myself while I'm running” (P19)* *“You have to put a lot more thought into it. You have to make sure you're either wearing the right kind of undies or you've got enough pads and yeah, how long you'll be out for and if you pad will hold if you've had too much water. So you're either dehydrated or peeing yourself” (P15)*

## Discussion

This qualitative descriptive study explored women’s experiences of exercising and playing sport with PF symptoms. Women across a range of ages, levels of sport participation and PF symptom type reported that their symptoms negatively impacted on their ability to participate in exercise/sport, and for many resulted in alterations to preferred mode of exercise or cessation entirely. As well as impact on participation, symptomatic women experienced a broad range of emotions whilst attempting to engage in exercise. Participants reported anxiety that their symptoms may become overt, frustration at the ‘rules’ that governed attempts to avoid symptoms and disappointment at losing the ability to participate in something they previously enjoyed. Exercising women employed a large range of coping strategies to minimise symptom provocation and allow ongoing participation; many of which felt restrictive and at times concerning for their health, i.e. restricting fluid intake. The degree of impact and ability to employ coping strategies were heavily influenced by the culture of the sport/exercise environment. Positive environments allowed women to feel supported and continue to participate, whereas negative environments created additional barriers to coping with symptoms and often resulted in disengagement.

A previous meta-ethnography on community dwelling women’s experiences of living with UI, found that women go to great lengths to hide and control their UI symptoms by avoiding social situations, limiting ‘risky’ activities and symptom hyper-vigilance [46]. However, sport/exercise activities are ‘risky’; they require exertion and create gravitational forces that place load on the PF. High-impact (jumping, tackling and running) and heavy lifting requirements of sport/exercise commonly provoke PF symptoms. Women with PF symptoms therefore face a unique challenge of attempting to engage in sports/exercise, whilst avoiding/managing symptoms.

One of the most common ways to avoid or manage symptoms is to stop exercising or modify exercise. A previous study by our group, found that 46% of women who experienced PF symptoms during exercise stopped participation due to their symptoms [5]. Women also reported modifying exercise to include lower impact, less frequent or intense forms of exercise [5]. The reasons women make the decision to stop or modify exercise has not been previously explored in depth. To date, qualitative data are largely drawn from studies reporting on impact on overall quality of life [22, 23, 26, 47], rather than focused on women engaging in exercise/sports settings. The rich qualitative data obtained in this study from exercising women, allows us to understand and develop strategies to address the challenges symptomatic female athletes experience within sports/exercise settings.

In the current study, women reported that exercising with PF symptoms is far more complex than simply reducing intensity of exercise or avoiding high-impact activities. To continue with exercise, women plan ahead, problem solve to address specific barriers posed by their symptoms or the exercise environment, and adapt when, where and with whom they exercise. However, women in our study reported a reluctance to participate in such group exercise settings/organised sport due to (1) fear that others may see, hear or smell their PF symptoms (2) feeling socially isolated and ‘different’ when they are unable to participate in symptoms provoking activities and (3) concerns/judgements from others that they are lazy or not putting in their best effort. National campaigns such as ‘Girls Make your Move’[48] and ‘Sport 2030’[49], are targeted at improving female representation in organised team sports, as women currently participate at lower rates than men [49]. Such health promotion activities are likely to be ineffective for women with PF symptoms unless their concerns are recognised. If we are to address women’s low levels of physical activity and participation in organised sport, we need strategies to address the unique challenges symptomatic women face to participation.

Community dwelling women report that living with UI evokes feelings of shame and humiliation; they are concerned that others will notice their symptoms in social settings and judge them unfavourably [46]. The current study builds on knowledge obtained from prior survey data that female athletes report embarrassment, fear, anxiety and frustration as a consequence of PF disorders [20]. Many of the same negative emotions were also reported by participants within our study and the basis for experiencing these emotions described. The mental health and social benefits of sport/exercise are well known [50]; however, for symptomatic women, rather than benefiting from the positive emotions associated with exercise, PF symptoms led to significant frustration and feelings of social isolation. Women expressed significant anxiety that what they are determined to keep private would become known. They felt frustrated that they had to be so hypervigilant of symptoms whilst exercising, missing their previous experiences that exercise had been spontaneous and relaxing. Women face a number of barriers to exercise [51]; for women already struggling to find the motivation to exercise, the additional barrier of experiencing PF symptoms may make engaging in sport/exercise seem insurmountable.

One of the primary frustrations expressed by participants was the number of detailed coping strategies that were needed to allow exercise/sport participation. Adding support to previous quantitative evidence, coping strategies reported included emptying the bladder on multiple occasions and fluid restriction [20]. Participants were aware of the dangers of not hydrating whilst exercising in hot climates but had to balance this against avoiding symptom provocation. Additionally, a primary concern raised by participants was that urinary/faecal leakage or pads may be visible to others when they are required to wear light coloured or ‘skimpy’ uniform. Sports uniform design for female athletes has generated conversation recently in relation to balancing sponsorship/organisation dress code requirements against athlete comfort and body image concerns [52]. Uniforms have been reported as a reason for ceasing sport participation in women particularly adolescent girls [53, 54]. Our findings provide additional rationale for future considerations of female athlete uniform requirements and the need to carefully consider design to allow symptom/pad concealment.

The culture of the sports environment was found to be an important factor that influenced symptomatic women’s ability to feel supported and cope with the physical and social impact of symptoms. Sports and exercise organisations can play an important role in creating a culture in which women’s health conditions can be discussed in a supportive, educative and normalised manner. Having role models that expressed a positive story and opened a forum for discussion of PF symptoms helped women feel less isolated and more hopeful about the future. Role models could be in the form of well-known female athletes publicly discussing their experiences or could take the form of health and exercise professionals demonstrating knowledge or lived experience. Health and exercise professionals can help to facilitate discussions around important women’s health topics and create an environment where they build rapport/trust, allowing disclosure of symptoms without judgement. Involving women in the codesign of acceptable screening and management practices within sport settings, could help to create an environment where women feel comfortable seeking help and exercise modifications to address PF symptoms, rather than dropping out of sport entirely.

### Strength and Limitations

The contribution this paper makes is that it is the first qualitative study focused on the experience of exercising with PF symptoms, addressing a paucity of evidence, identified in previous reviews [20, 21]. Use of open-ended, interview questions in a private one–one setting allowed us to gain rich, detailed understanding of symptomatic exercising women’s experiences. Due to the COVID-19 pandemic, one–one interviews were conducted via Zoom online rather than in-person. The researchers did not perceive a negative effect on depth of information obtained via this method; interviews were of sufficient length, participants spoke freely of their experiences and being online allowed involvement of participants from Australia wide.

Previous quantitative data have reported on the impact of UI symptoms on exercise; participants in our study had experienced a range of PF disorders including POP, AI and pelvic pain symptoms allowing a better representation of the impact that a range of PF symptoms may have on exercise/sport. To further reflect that participants represented the lived experience of women with PF disorders, participants also reported a breadth of PF symptom severity and bother. The child bearing and post-menopausal years are a key period for PF symptom onset, however women who are nulliparous also experience symptoms whilst exercising [10]. Many of the previous qualitative studies that included narrative on the impact of PF symptoms on QOL (including exercise) focused on post-natal or post-menopausal women only [23, 24, 26]. Three participants in our study were nulliparous and spoke of the unique stigma associated with experiencing PF symptoms without having given birth.

We acknowledge a limitation of the study is that despite targeted recruitment strategies, there were no participants < 26 years of age in our study. Difficulties recruiting younger women to the study may be due to reluctance to discuss intimate symptoms; PF symptoms are considered common after childbirth but the high prevalence in younger women also is not commonly acknowledged and may increase the perception of PF disorders as a taboo topic in this age group. Our study contained two elite/semi-elite athletes, which is representative of the proportion of female athletes who participate at an elite level of competition in sport. However, it is likely that elite athletes face unique and additional challenges to exercising with PF symptoms such as impact on sponsorship, finances and performance and therefore further qualitative studies in this group/setting are warranted. All interviews were conducted in English which limited involvement from non-English speaking participants. As with all qualitative data analysis, findings of this study could be influenced by researcher preconceptions. We attempted to enhance the credibility of our findings by: (1) use of a research journal to record reflections on researcher assumptions and perceptions and (2) open and regular discussion on the interpretation of data by the research team who represented varied clinical/research expertise and come from diverse cultural backgrounds. We checked our data summaries with participants on two occasions providing an opportunity for feedback from participants on findings, and selected participant quotations from the whole sample.

## Conclusion

The findings of this study demonstrate that PF symptoms adversely impact on women’s ability to be able to participate in sport/exercise. Attempting to exercise with PF symptoms caused negative emotions such as embarrassment, fear and frustration that contributed further to the challenges of maintaining participation. Women used detailed and onerous coping strategies to minimise symptom provocation but found this reduced the spontaneity and social benefits of participation. The culture of the sports/exercise environments influenced whether women felt supported to participate or discouraged and unable to continue. Sports and exercise organisations should be aware of the impact of PF symptoms on sports/exercise participation and consider strategies such as screening, education and management of symptoms with the aim of creating a positive and supportive experience for symptomatic women and reducing cessation in participation.

## Supplementary Information

**Additional file 1**: Email sent to participants prior to research summarising topics for discussion.

## Data Availability

The datasets generated and/or analysed during the current study are not publicly available due to the need to maintain privacy of information; data are interview transcripts, but are available from the corresponding author on reasonable request.

## Abbreviations

PF: Pelvic floor
UI: Urinary incontinence
SUI: Stress urinary incontinence
UUI: Urgency urinary incontinence
MUI: Mixed urinary incontinence
AI: Anal incontinence
POP: Pelvic organ prolapse
P: Participant
