# Supplementary material for: Experience of Playing Sport or Exercising for Women with Pelvic Floor Symptoms: A Qualitative Study
Source: Sports Med Open. 2023 Apr 25;9:25. doi: 10.1186/s40798-023-00565-9 (PMC10127961; doi:10.1186/s40798-023-00565-9)
Supplement: Supplementary file 1 — Additional file 1: Email sent to participants prior to research summarising topics for discussion. [file 40798_2023_565_MOESM1_ESM.pdf]

Experience of Playing Sport or Exercising for Women with Pelvic Floor Symptoms: A Qualitative Study

Sports Medicine - Open

Jodie G. Dakic (PT) [a,i]
Jean Hay-Smith (PhD) [b]
Kuan-Yin Lin (PT, PhD) [c,d]
Jill Cook (PT, PhD) [e]
Helena C. Frawley (PT, PhD) [f,g,h]

a. Department of Physiotherapy, Monash University, 47 - 49 Moorooduc Highway Frankston, Victoria, 3199, Australia, jodie.dakic@monash.edu
b. Rehabilitation Teaching and Research Unit, Department of Medicine, University of Otago, Wellington, PO Box 7343, Wellington South 6242, New Zealand.
c. Department of Physical Therapy, National Cheng Kung University, No.1, Ta-Hsueh Road, Tainan 701, Taiwan.
d. Institute of Allied Health Sciences, College of Medicine, National Cheng Kung University, No.1, Ta-Hsueh Road, Tainan 701, Taiwan.
e. La Trobe Sport and Exercise Medicine Research Centre, La Trobe University, Plenty Rd &, Kingsbury Dr, Bundoora, Victoria, 3086, Australia.
f. Melbourne School of Health Sciences, The University of Melbourne, 161 Barry St., Parkville VIC 3010, Australia.
g. Allied Health Research, The Royal Women's Hospital, Parkville, VIC 3052, Australia
h. Allied Health Research, Mercy Hospital for Women, Studley Rd, Heidelberg, VIC 3084, Australia
i. Department of Physiotherapy, The University of Melbourne, 161 Barry St, 3010, Parkville, Australia

---

Supplementary 1: Email sent to participants prior to research summarising topics for discussion

Dear Participant,

Thank-you for volunteering to participate in our research project titled: **Women's experiences of and preferences for pelvic floor symptom screening in sports/exercise settings.**

Thank-you also for completing the consent form and short survey. You have met the inclusion criteria for the study and I look forward to setting up an interview at a time that is convenient for you. The interview will take approximately one hour and will be conducted online over Zoom.

The interview will include a discussion on the following topics:

- Your experience and disclosure of pelvic floor symptoms in a sport/exercise setting
- Your experience of being screened for pelvic floor symptoms within sports/exercise settings
- Your perceptions and preferences for pelvic floor symptom screening in sport/exercise

It is ideal if we conduct the interview at a time and location when you have privacy to allow you to discuss the topics freely. What days and times might suit you over the next week or two to conduct the interview?

Once I have received your reply, I will schedule a time that works for both of us. It can be an evening or a weekend if that works better for you. I will then send you a zoom calendar invitation and link to access the meeting at the time we nominate.

Please let me know if you have any questions. I look forward to meeting with you.

Kind Regards
Jodie Dakic
